# Dysregulation of EZH2/miR-138-5p Axis Contributes to Radiosensitivity in Hepatocellular Carcinoma Cell by Downregulating Hypoxia-Inducible Factor 1 Alpha (HIF-1*α*)

**DOI:** 10.1155/2022/7608712

**Published:** 2022-08-29

**Authors:** Bing Bai, Ying Liu, Xue-Mei Fu, Hai-Yan Qin, Gao-Kai Li, Hai-Chen Wang, Shi-Long Sun

**Affiliations:** ^1^NHC Key Laboratory of Radiobiology, School of Public Health, Jilin University, Changchun, Jilin 130021, China; ^2^Department of Toxicology, School of Public Health, Jilin University, Changchun, Jilin 130021, China; ^3^Jilin Women and Children Health Hospital, Changchun, Jilin 130061, China; ^4^Department of Plastic Surgery, China-Japan Union Hospital of Jilin University, Changchun, Jilin 130031, China

## Abstract

Enhancer of zeste homolog 2 (EZH2) is a histone methyltransferase involved in cell proliferation, invasion, angiogenesis, and metastasis in various cancers, including hepatocellular carcinoma (HCC). However, the role and molecular mechanisms of EZH2 in HCC radiosensitivity remain unclear. Here, we show that EZH2 is upregulated in HCC cells and the aberrantly overexpressed EZH2 is associated with the poor prognosis of HCC patients. Using miRNA databases, we identified miR-138-5p as a regulator of EZH2. We also found that miR-138-5p was suppressed by EZH2-induced H3K27me3 in HCC cell lines. MiR-138-5p overexpression and EZH2 knockdown enhanced cellular radiosensitivity while inhibiting cell migration, invasion, and epithelial-mesenchymal transition (EMT). Analysis of RNA-seq datasets revealed that the hypoxia-inducible factor-1 (HIF-1) signaling pathway was the main enrichment pathway for differential genes after miR-138-5p overexpression or EZH2 knockdown. Expression level of HIF-1*α* was significantly suppressed after miR-138-5p overexpression or silencing of EZH2. HIF-1*α* silencing mitigated resistance of HCC cells and inhibited EMT. This study establishes the EZH2/miR-138-5p/HIF-1*α* as a potential therapeutic target for sensitizing HCC to radiotherapy.

## 1. Introduction

Hepatocellular carcinoma (HCC) is one of the malignant tumors with high mortality in the world [1]. Surgical resection is the first-line treatment for patients with early-stage HCC [2], but those with advanced-stage disease are unsuitable for surgery [3]. In recent years, advanced radiation therapy, such as stereotactic body radiation therapy (SBRT), has shown high efficiency in suppressing locoregional HCC. Such treatment necessitates the development of radiation sensitizers to enhance the therapeutic ratio and clinical outcomes.

Enhancer of zeste homolog 2 (EZH2), the catalytic subunit of polycomb repressor complex 2 (PRC2), specifically trimethylates histone 3 at lysine 27 (H3K27me3) and functions as a transcriptional repressor via chromatin modification [4]. Several studies have reported the utility of EZH2 as a potential therapeutic target for HCC owing to its marked upregulation in HCC and correlation with poor prognosis of HCC [5–7]. The HCC-promoting role of EZH2 stems from its regulation on epigenetic silencing of signal transducer and activator of transcription 3 (STAT3), TGF-beta, and Wnt pathway via inhibitors, such as axis inhibition protein 2 (AXIN2) and prickle planar cell polarity protein 1 (PRICKLE1) [8–10]. Although the lysine methyltransferase activity of EZH2 is considered majorly related to its cancer regulatory role, EZH2 may also regulate HCC through other mechanisms [11, 12]. Silencing EZH2 has been found to effectively inhibit malignant behaviors of tumor cells, which has hence spurred tremendous efforts to find novel and efficient EZH2 inhibitors [13, 14]. One of such inhibitors is miR-138-5p, which is a noncoding RNA that can suppress the progressive behavior of HCC cells [15, 16].

Hypoxia is a common tumor biomarker, and hypoxic tumor cells activate stress response pathways to adapt to the low oxygen level. Tumor cells under hypoxia adapt via altering their gene expression to facilitate their survival and even proliferation. These processes contribute to the cancer cells' aggressive phenotype and resistance to therapy [17]. Reduced sensitivity of hypoxic tumor cells to radiotherapy is one of the factors contributing to poor clinical outcomes [18]. Chen et al. found that hypoxia increases the expression of HIF-2*α* via the PI3K-AKT-mTOR pathway, which contributes to HCC progression [19].

The hypoxia-inducible factor (HIF) is a heterodimer composed of three different oxygen-sensitive HIF*α* subunits (HIF-1*α*, HIF-2*α*, and HIF-3*α*) and a constitutively expressed *β*-subunit. HIF-1*α* and HIF-2*α* heterodimers function as transcriptional activators of oxygen-regulated target genes [20]. Transcriptional regulation of different genes by HIF-1*α* in hypoxic cells may contribute differentially to the malignant phenotype in cancer cells. Studies have shown that inhibition of HIF-1 leads to metabolic changes that enhance radiation therapy efficacy [21]. Yang et al. reported that HIF-1 downregulation by siRNA enhanced the radiosensitivity of hypoxic HCC cells [22], suggesting that the combined treatment using HIF-1 inhibitors and radiotherapy enhances the anticancer effect on HCC.

Therefore, the present study strived to investigate the role of the EZH2/miR-138-5p axis on the radiosensitivity of HCC. Our study provided evidence that EZH2 expression is regulated by miR-138-5p, which in turn is epigenetically downregulated by EZH2 through a double-negative feedback loop in HCC cells. Moreover, EZH2/miR-138-5p negative correlation is significantly associated with radiosensitivity in HCC patients through inhibiting HIF-1*α*.

## 2. Materials and Methods

### 2.1. Cell Culture and Transfection

Human liver cancer cell lines HepG2 and Hep3B, as well as HEK-293 T cells, were purchased from Cell Bank of the Chinese Academy of Sciences (Shanghai, China), cultured in DMEM/MEM medium supplemented with 10% fetal bovine serum (FBS) and 1% penicillin/streptomycin, and placed at 37 °C in a humidified incubator containing 5% CO_2_.

Mimic of miR-138-5p, S: AGCUGGUGUUGUGAAUCAGGCCG, AS: GCCUGAUUCAC AACACCAGCUUU, the mimic negative control (NC), S: UUCUCCGAACGUGUCACGUTT, AS: ACGUGACACGUUCGGAGAATT, and siRNA of HIF-1*α* (ACCCUAACUAG CCGAGGAAGAATT) were acquired from GenePharma Co., Ltd. (GenePharma, Shanghai, China). Their sequences are shown in Table 1. Lentivirus vector and EZH2-shRNA/vector plasmid were used to build HepG2-shEZH2/HepG2-vector model.

Cells were sowed into 6-well plates (about 2 × 10^6^ cells/well) and transfected with aforementioned nucleotides or plasmids when confluence reached 60-70% using Sage LipoplusTM (Sagecreation, Beijing, China) referring to manufacturer's recommendations.

### 2.2. Real-Time Quantitative PCR

Total RNA was extracted using the Trizol (Thermo Fisher Scientific, Waltham, MA, USA). For mRNA quantification, cDNA was synthesized using the PrimeScript RT Reagent Kit (TaKaRa, Shiga, Japan) and detected using the SYBR Green PCR Mastermix (Solarbio, Beijing, China) following the manufacturer's protocols. For miRNA quantification, cDNA was synthesized and measured using the Hairpin-it microRNA and U6 snRNA Normalization RT-qPCR Quantitation Kit (GenePharma, Shanghai, China). The primers used for hsa-miR-138-5p and U6 were obtained from GenePharma. Primer sequences are listed in Table 1.

### 2.3. Bioinformatics Analysis

We performed expression analyses of genes using TCGA data through the ULCAN portal (http://ualcan.path.uab.edu/index.html) [23]. And the survival graphs were downloaded and processed from the Kaplan-Meier plotter portal (http://kmplot.com/analysis) [24]. We searched for targets of has-miR-138-5p using several target prediction algorithms databases, including PicTar, miRanda, miRTarBase, and TargetScan.

### 2.4. Dual-Luciferase Reporter Gene Assay

HEK-293 T cells (1 × 10^4^ cells per well) were seeded in 96-well culture plates and co-transfected with 200 ng EZH2-3′UTR or EZH2-3′UTR-Mut vectors and miRNA mimic or NC (10 pM) using LipoPlus™ Reagent (Sagecreation, Beijing, China). Forty-eight hours later, HEK-293 T cells were collected, and the luciferase activity changes of miR-138-5p on EZH2-3′UTR were measured using a dual-luciferase assay kit (Promega, Madison, WI, USA) by a luminometer fluorescence detector (BioTek, Biotek Winooski, Vermont, USA).

### 2.5. Western Blot

Total protein of cells was extracted by using RIPA buffer containing protease inhibitors (Solarbio, Beijing, China). Protein concentration was quantified using Enhanced BCA Protein Assay Kit (Beyotime Biotechnology, Shanghai, China). The total proteins (30 *μ*g for each) were separated using 10% SDS-PAGE and transferred to polyvinylidene fluoride membranes. Following blocking with 5% skimmed milk at room temperature for 1 h, the membrane was incubated primary antibodies at 4 °C overnight. After incubation with primary antibodies, membranes were washed with TBST followed by HRP-conjugated secondary antibodies for 1 hour at room temperature. These bands were detected using ECL solution (Solarbio, Beijing, China).

### 2.6. Clonogenic Assay

Seed a predetermined number of cells in a 6-well culture plate. After 24 hours, cells were treated with a series of radiation doses (0, 2, 4, 6, and 8 Gy) (Model X-RAD320iX; Precision X-Ray, Inc., North Branford, CT, USA). After incubating these cells at 37 °C for 7-10 days, they were washed twice with PBS, fixed with formaldehyde and finally stained with crystal violet solution. Count the number of colonies containing ≥50 cells, colony formation efficiency = (number of colonies/number of inoculated cells) × 100%. Survival fraction (SF) was calculated by normalizing the clonogenic efficiency of the control group. We fit cell survival curves according to standard linear quadratic (LQ) models using GraphPad Prism 5 (GraphPad Software, LaJolla, CA, USA).

### 2.7. Wound Healing Assay

Wound healing assay is used to detect cell migration ability. The cells are spread on 6-well plates and transfected when the cells reach at 80% confluence. After 48 h of transfection, scrape the cells with a 200 *μ*l pipette tip. Use DMEM or MEM supplemented for cell culture to reduce the potential impact of cell proliferation on the final result. Fixed point image acquisition was performed at 0 h, 24 h, and 48 h, respectively.

### 2.8. Transwell Invasion Assay

Transwell invasion assay detects cell invasion ability. After 48 h of transfection, cells were resuspended in serum-free DMEM or MEM and transferred to the transwell chamber. The total volume of liquid in the chamber was 200 *μ*l, and 550 *μ*l of complete medium was injected out of the chamber. After 24 h incubation, the culture medium was discarded, washed twice with PBS, fixed with 4% paraformaldehyde for 20 min, and washed twice with PBS. Finally, stain with 0.1% crystal violet for 30 min, wash twice with PBS, and observe the cells under a microscope.

### 2.9. Flow Cytometry

After 48 hours of transfection, the logarithmic growth phase cells were centrifuged at 1500 rpm for 5 minutes, and the supernatant was discarded. After adding PBS, centrifuge at 1500 rpm for 5 minutes. Repeat the operation. After that, cells should be fixed in ice-cold 70% ethanol for at least 1 h. Using PI Solution (Meilunbio Biotech, Dalian, China), prepare a cell suspension with a cell concentration of 1 × 10^6^ cells/ml using the prepared 1 × PI Solution. After 30 minutes of incubation in the dark, it was detected within 1 hour. For the test of apoptosis, annexin V-FITC (Meilunbio, Biotech, Dalian, China) binding solution was added before PI solution, and it was detected within 1 hour. 2′,7′-dichlorofluorescein diacetate (DCFH-DA, Solarbio, Beijing, China) was used for detection of intracellular ROS.

### 2.10. Chromatin Immunoprecipitation (ChIP) Assay

ChIP was performed using a ChIP-IT® Express Enzymatic Shearing Kit (Active Motif, Carlsbad, CA, USA) on cells. Briefly, protein-DNA complexes were cross-linked by incubating cells in 1% formaldehyde-containing medium for 10 min. Cell pellets were resuspended in lysis buffer with protease inhibitors. Cells were digested by restriction endonuclease, in order to shear DNA to fragments about 200 bps. Samples were taken at this point as positive controls in the RT-qPCR reaction (input chromatin). Next, cells were incubated with monoclonal antibody against H3K27me3 (Abcam, Cambridge, MA, USA) overnight at 4 °C. Human purified IgG was used as control antibody (Immunoway, Newark, Delaware, USA). DNA-protein complexes were collected using Protein A/G Magnetic beads (MedChem Express, Monmouth Junction, NJ, USA), followed by washing, elution, and reverse cross-linking. DNAs were purified with Chromatin IP DNA Purification Kit (Active Motif, Carlsbad, CA, USA). Recovered DNAs were resuspended in TE buffer and were later analyzed by RT-qPCR (Primers are shown in Table 1).

### 2.11. RNA-Seq Analysis

For RNA-seq, we divided HepG2 cells into four groups, transfected with shEZH2/vector plasmid or miR-138-5p mimic/NC. RNA isolation, library construction, and sequencing were performed on a BGISEQ-500 (Beijing Genomic Institution, BGI). The sequencing data analysis, including heat map clustering, Venn diagram creation, gene ontology (GO) analysis, and Kyoto Encyclopedia of Genes and Genomes (KEGG) analysis, was performed using BGI Dr. Tom 2.0.

### 2.12. Statistical Analysis

SPSS 24.0 and GraphPad Prism 5 was applied. All measurement data are expressed as mean ± standard deviation (SD) or standard error of the mean (SEM), with at least three independent replications. The comparison between two groups was conducted by independent-samples *t* test. Kaplan-Meier (K-M) curve was used to analyze the relationship between the expression of miR-138-5p, EZH2, and the 5-year survival rate of HCC patients, and the log rank test was used for variance analysis. *p* value less than 0.05 was considered to indicate statistical significance: ∗*p* < 0.05 and ∗∗*p* < 0.01.

## 3. Results

### 3.1. Expression Levels of EZH2 and miR-138-5p Are Associated with HCC Prognosis

**Figure 1:**
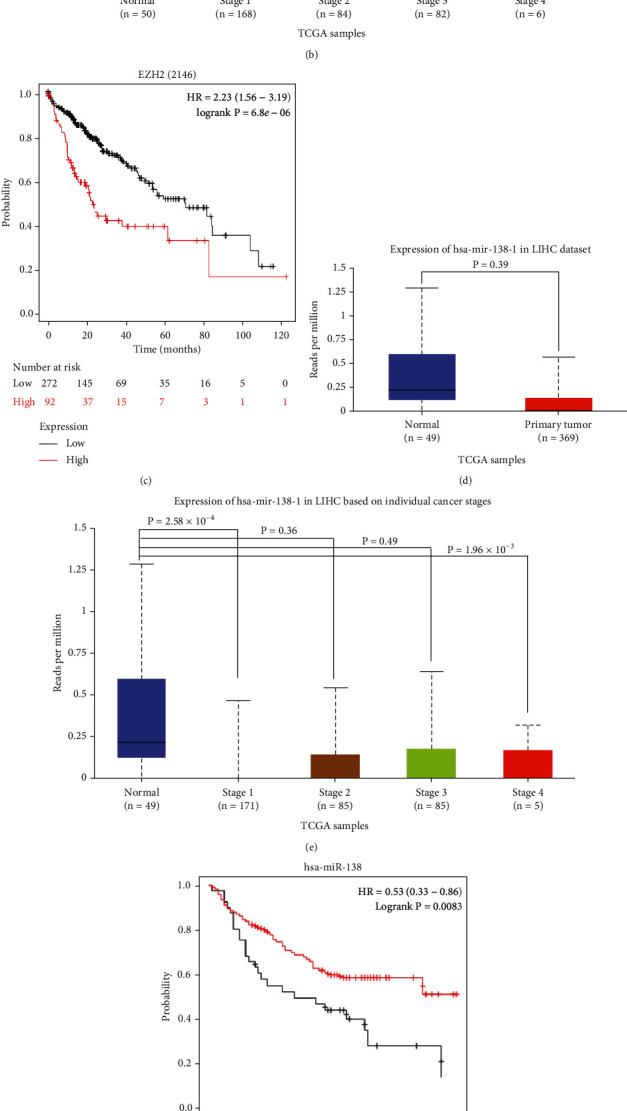
Expression of EZH2, miR-138-5p in subgroups of liver cancer patients with different prognoses and validation in vitro. (a) The plot shows EZH2 mRNA expression level between HCC specimens and liver tissue. (b) The plot shows EZH2 mRNA expression level in grades 1-4 HCC patients. (c) EZH2 expression was correlated with poorer OS in the HCC cohort of the Kaplan Meier plotter database. (d) The plot shows miR-138-5p expression level between HCC specimens and liver tissue. (e) The plot shows miR-138-5p expression levels in grades 1-4 HCC patients. (f) The level of miR-138-5p expression was correlated with poorer OS in the HCC cohort of the Kaplan Meier plotter database. (g) EZH2 mRNA expression was measured in five HCC cells (HepG2, Hep3B, Huh-7, PLC/PRF/5, and SMMC-7721) and immortalized human liver cell line L-02. (h) EZH2 protein expression was measured in five HCC cells (HepG2, Hep3B, Huh-7, PLC/PRF/5, and SMMC-7721) and immortalized human liver L-02 cell line. (i) Level of miR-138-5p expression was tested in five HCC cells (HepG2, Hep3B, Huh-7, PLC/PRF/5, and SMMC-7721) and immortalized human liver cell line L-02. ^∗^*p* < 0.05, ^∗∗^*p* < 0.01 (compared to L-02).

In order to explore the role of miR-138-5p and EZH2 in HCC, we compared their expression in HCC specimens and adjacent nontumor liver tissue in The Cancer Genome Atlas (TCGA) dataset. Our results showed that the expression of EZH2 mRNA in HCC primary tumor tissue (*n* = 371) was significantly higher than that in liver tissue adjacent to the tumor (*n* = 50) (Figure 1(a)). Expression of EZH2 was found to be significantly higher in tumors of higher grades compared to normal tissues, suggesting that EZH2 may be related to the progression of HCC (Figure 1(b)). Then, the Kaplan Meier (KM) plotter online tool was used to establish the relationship between EZH2 expression and the survival outcomes of HCC [25], which revealed that HCC patients with a higher level of EZH2 had a poorer prognosis (OS: HR = 2.23, 95%CI = 1.56 to 3.19, *p* < 0.001) (Figure 1(c)).

To explore whether miR-138-5p is associated with EZH2 overexpression in HCC patients, we analyzed the level of has-miR-138-1, one member of the pre-miR-138-5p family, in the TCGA database. Interestingly, has-miR-138-1 was significantly downregulated in HCC specimens compared to normal tissues (Figure 1(d)). Has-miR-138-1 was pronouncedly lower expressed in patients with the grade 1 and grade 4 HCC, but not grade 2 and grade 3 HCC (Figure 1(e)). Similarly, survival analysis showed that lower level of miR-138-5p is associated with a poorer prognosis (OS: HR = 0.53, 95%CI = 0.33 to 0.86, *p* < 0.01) (Figure 1(f)). To explore further the biological function of miR-138-5p and EZH2 in HCC cells, we examined their expression pattern in HCC cell lines, using the human liver cell line as a control. Consistent with the bioinformatics analysis, the expression levels of EZH2 protein and mRNA in five HCC cell lines, including HepG2 and Hep3B, were significantly higher than that in the immortalized human liver L-02 cell line (Figures 1(g) and 1(h)). Conversely, the level of miR-138-5p is lower in HCC cells than that in L-02 cells (Figure 1(i)).

### 3.2. In HCC Cells, EZH2 Is a Direct Target of miR-138-5p, which Is Epigenetically Regulated by EZH2 in H3K27me3-Dependent Way through a Negative Feedback Loop

**Figure 2:**
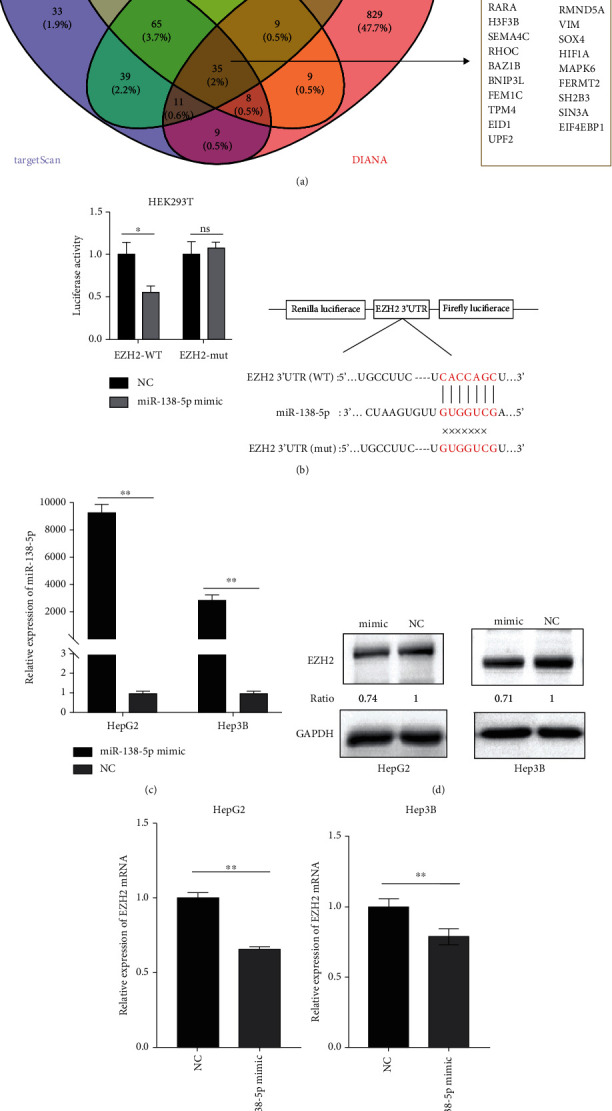
EZH2 is a direct target of miR-138-5p. (a) Venn diagram indicating the intersection of the number of target genes among the above four databases. (b) HEK293T cells were co-transfected with EZH2 3′UTR-wt or EZH2 3′UTR-mut with miR-138-5p mimics or NC. Following 48 h of transfection, luciferase activity was measured. (c) HepG2 and Hep3B were transfected with miR-138-5p mimic or NC for 24 h. The expression of miR-138-5p was determined by RT-qPCR analysis. The expression of U6 was used as an internal control. (d–e) HepG2 and Hep3B were transfected with miR-138-5p mimic or NC for 48 h. The expression of EZH2 was determined by Western blotting and RT-qPCR analysis. GAPDH was used as a loading control. ^∗^*p* < 0.05; ^∗∗^*p* < 0.01 (compared to NC group or vector group); ns, no significance.

To explore whether miR-138-5p are associated with EZH2 aberrant overexpression in HCC cells, we searched for the targets of miR-138-5p using a variety of target prediction algorithm databases, including PicTar, miRanda, TargetScan, and DIANA. After taking the overlapping target molecules, 35 genes, including EZH2, are found to be the common target genes of miR-138-5p (Figure 2(a)). Comprehensive bioinformatics analysis was carried out, which showed that miR-138-5p had a negative correlation with EZH2 expression level. The luciferase reporter assay also confirmed that EZH2 is a direct target of miR-138-5p (Figure 2(b)). HEK-293 T cells were also transfected with the mutant or wild-type EZH2-3′UTR luciferase reporter vectors together with miR-138-5p mimic or negative control, which showed that miR-138-5p overexpression significantly reduced wild-type EZH2-3′UTR reporter luciferase activity, but not that of the mutant-3′UTR reporter. This data indicated that miR-138 could directly target the EZH2 3′UTR. To further evaluate the inhibitory effect of miR-138-5p on EZH2, HepG2 and Hep3B cells were transfected with miR-138-5p mimic and mimic NC, and the overexpression of miR-138-5p was verified by RT-qPCR (Figure 2(c)). In HepG2 and Hep3B cells, the mRNA and protein expression of EZH2 were significantly inhibited by miR-138-5p overexpression (Figures 2(d) and 2(e)).

**Figure 3:**
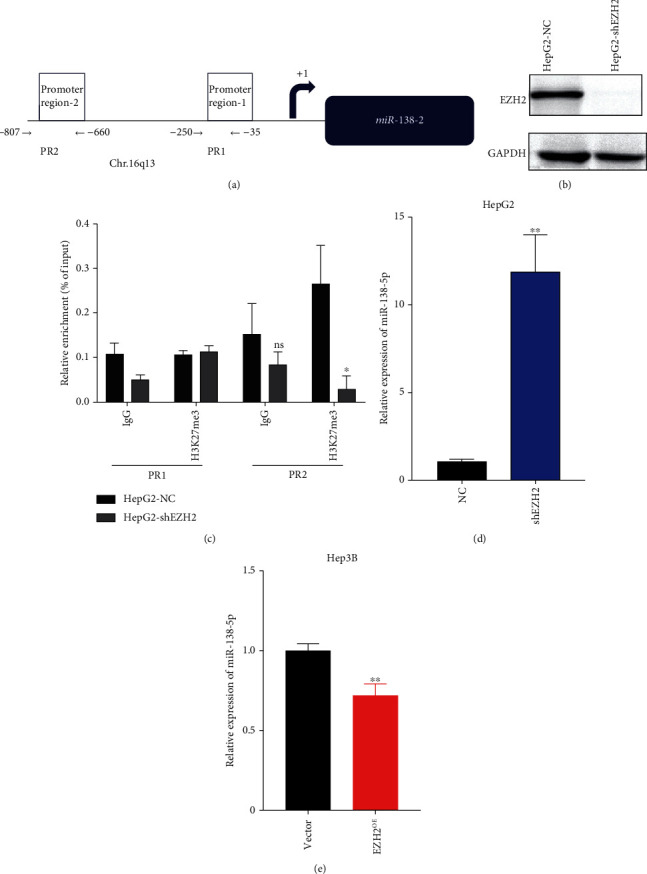
miR-138-5p is epigenetically regulated by EZH2. (a) Binding sites of primers used for the analysis of chromatin immunoprecipitation (ChIP) are indicated relative to the transcription start site. (b) Expression of EZH2 in HepG2-shEZH2 and HepG2-NC groups. (c) The HepG2-NC and HepG2-shEZH2 were cultured and subjected to ChIP using H3K27me3 antibodies, or as control, normal human IgG. The precipitated chromatin was analyzed by RT-qPCR for the abundance of region PP1&PP2 upstream of the miR-138-5p gene. Values were normalized to chromatin levels in 1% input samples. (d) Total RNA of HepG2-shEZH2 was isolated from cells and the miR-138 level was quantified by RT-qPCR (E) Hep3B was treated with EZH2 overexpression plasmid and total RNA was isolated from cells. The miR-138-5p level was quantified by RT-qPCR. ^∗^*p* < 0.05; ^∗∗^*p* < 0.01 (compared to NC group or vector group).

Next, we explored the molecular mechanism underlying miR-138-5p downregulation in HCC cells. We designed two primer pairs to amplify the two promoter regions of the miR-138 gene, i.e., promoter region 1(PR1, from − 35 to − 250 bp) and promoter region 2 (PR2, from − 660 to − 807 bp) upstream. Chromatin immunoprecipitation (ChIP) was performed to assess the repressive histone marker H3K27me3 (Figure 3(a)). To further study the regulatory role of EZH2 on miR-138-5p, the EZH2 gene was silenced with shRNA in HepG2 (Figure 3(b)). Furthermore, ChIP assays with H3K27me3 antibody showed that it was less occupied in the upstream regions of PR2 in HepG2-shEZH2 (Figure 3(c)). Thus, H3K27me3 upregulation induced by EZH2 may contribute to miR-138-5p suppression. These data confirmed that miR-138-5p was upregulated in HepG2-shEZH2 (Figure 3(d)). Conversely, cells overexpressing EZH2 showed a reduced level of miR-138-5p (Figure 3(e)). Taken together, these results indicate that miR-138-5p and EZH2 can form a negative feedback loop that regulates the phenotypes of HCC.

### 3.3. Overexpression of miR-138-5p or Knockdown of EZH2 Enhances Radiosensitivity of HCC Cells

**Figure 4:**
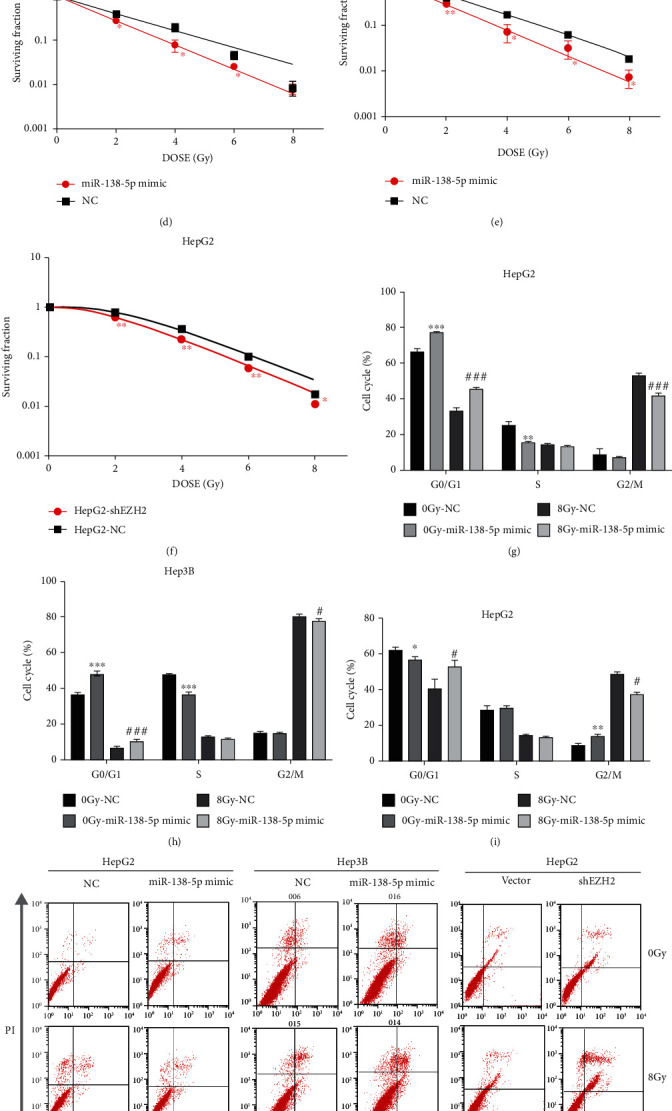
Overexpression of miR-138-5p by mimic or knockdown of EZH2 enhances radiosensitivity of HCC cells. (a–c) Experiments results of three groups of cells' clone formation experiments. (d–f) The SF of three groups of cells. (g–i) HepG2 and Hep3B cells were transfected with miR-138-5p mimic/NC or shEZH2/vector plasmid before exposure to 8 Gy radiation. After 24 h, cells were harvested for cell-cycle analysis. (j–m) HepG2 and Hep3B cells were transfected with miR-138-5p mimic/NC or shEZH2/vector plasmid before exposure to 8 Gy radiation. After 24 h, cells were harvested for apoptosis analysis. ^∗^*p* < 0.05; ^∗∗^*p* < 0.01 (compared to NC or vector group in HepG2); ^#^*p* < 0.05; ^##^*p* < 0.01 (compared to NC group after exposed to 8Gy).

To explore miR-138-5p's impact on HCC cells' radiosensitivity, clonogenic assays were performed after exposure to X-ray of different doses (0, 2, 4, 6 or 8 Gy). HepG2 and Hep3B cells in the logarithmic growth phase were divided into the miR-138-5p mimic group and NC group, each group with three repeated culture holes. Radiotherapy reduced the survival of the cells, as measured by survival fractions (SF), in both cell lines in a dose-dependent manner. However, cancer cells transfected with miR-138-5p mimic demonstrated a more significant decrease in SF compared to the NC group in all ranges of absorbed dose, and the cell survival curves decreased markedly (Figures 4(a)–4(d)). In the miR-138-5p mimic group, the radiation dose required to reduce the fraction of surviving cells to 37% of its previous value (D0 in radiation biology). The D0 dose for HepG2 cells decreased from 2.26 to 1.57 Gy and decreased from 1.77 to 1.42 Gy in Hep3B cells. Knocking down the EZH2 gene also achieved similar effects, i.e., the D0 dose for HepG2-shEZH2 decreased from 1.64 to 1.59 Gy (Table 2). These data suggest that the overexpression of miR-138-5p may significantly enhance the radiosensitivity of HCC cells. Various anticancer drugs are known to inhibit tumor cell division and induce cell death through cell cycle arrest, which prompted us to investigate whether the role of EZH2/miR-138-5p axis in enhancing radiosensitivity is facilitated by induced cell cycle arrest. After treating HCC cells with miR-138-5p mimic for 48 h, both HepG2 and Hep3B cells showed cell cycle arrest at the G0/G1 stage (Figures 4(g) and 4(h)). Compared to the NC group, the population of cells at the G0/G1 stage increased by 10.79% ± 1.07% in HepG2 cells and 4.17% ± 1.15% in Hep3B cells, respectively. In particular, the G0/G1 cycle arrest further increased in HepG2 after radiation treatment. Similarly, the HepG2-shEZH2 group also showed significantly increased G0/G1 arrest after radiation treatment (Figure 4(i)).

Radiation-induced apoptosis is one of the major mechanisms of cell death. Hence, we also analyzed how dysregulation of EZH2/miR-138-5p axis contributed to the apoptosis of HCC cells (Figure 4(j)). Compared to the NC group, treatment with miR-138-5p mimic significantly increased the number of AnnexinV-FITC positive cells in HepG2 and Hep3B cells after radiation (Figures 4(k) and 4(l)). Consistently, the apoptosis rate of the HepG2-shEZH2 group was higher than that of the HepG2-vector group after radiation (Figure 4(m)).

### 3.4. Overexpression of miR-138-5p or Knockdown of EZH2 Inhibits Radiation-Induced Cell Migration and Invasion via Suppressing EMT

**Figure 5:**
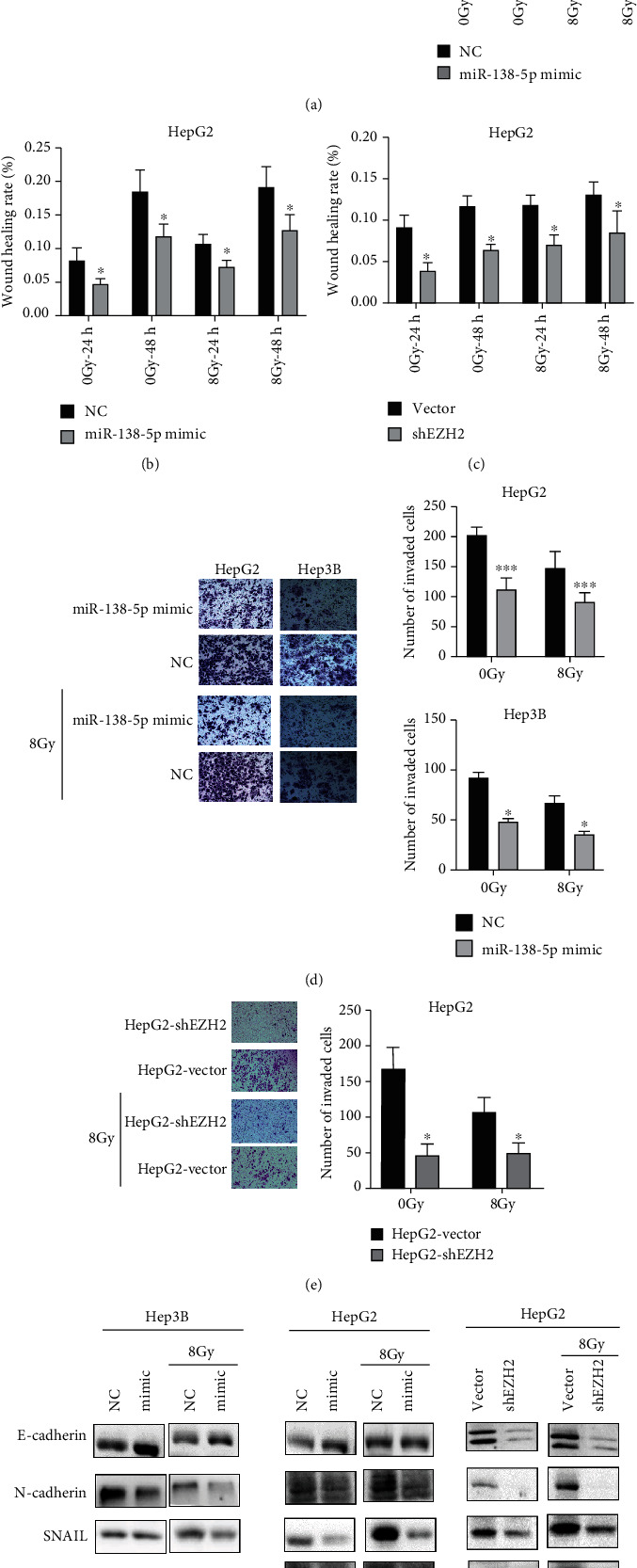
Overexpression of miR-138-5p or knockdown of EZH2 inhibits radiation-induced migration and invasion via EMT. (a–c) Wound healing assays of Hep3B and HepG2 cells infected with miR-138-5p mimic/NC or shEZH2/vector plasmid after radiation. (d–e) Transwell assays of Hep3B and HepG2 cells infected with miR-138-5p mimic/NC or shEZH2/vector plasmid after radiation. (f) The effect of miR-138-5p mimic or knockdown of EZH2 on the expression of EMT-related molecules by Western blotting. ^∗^*p* < 0.05 (compared to NC group or the vector group).

Given that radiation paradoxically promotes tumor cell migration and invasion while exerting cytotoxic effects, we explored whether the EZH2/miR-138-5p axis plays an important part in inhibiting cell migration and invasion while augmenting cellular radiosensitivity. In the mimic group, the relative wound healing rate was significantly reduced compared with the NC groups (Figures 5(a) and 5(b)). The high expression of miR-138-5p was also shown to inhibit the migration ability after being exposed to 8Gy radiation. The migratory ability of the HepG2-shEZH2 group was similar to that of the mimic group (Figure 5(c)). Knockdown of EZH2 also inhibited the migratory ability of HepG2 after radiation. As shown in Figures 5(d) and 5(e), overexpression of miR-138-5p or inhibition of EZH2 expression significantly decreased the migration and the invasion rate in HCC cells after radiation.

Since EMT is a putative molecular mechanism that drives tumor migration and invasion, we explored whether the EZH2/miR-138-5p axis is involved in EMT by analyzing levels of EMT markers including E-cadherin, N-cadherin, vimentin, and Snail. Our results indeed showed that the protein level of E-cadherin was significantly increased, which means adherens junction between cells was decreased. The protein levels of N-cadherin and vimentin were pronouncedly reduced in HepG2 and Hep3B cells after mimic or shEZH2 treatment, even after radiation, suggesting attenuated EMT (Figure 5(f)). Snail, a transcription factor that activates EMT, is also decreased after overexpressing miR-138-5p or inhibiting EZH2. Taken together, these results implicated that dysregulation of EZH2/miR-138-5p axis can suppress EMT in HepG2 and Hep3B cells.

### 3.5. Dysregulation of EZH2/miR-138-5p Axis Affects the Expression of HIF-1*α*

**Figure 6:**
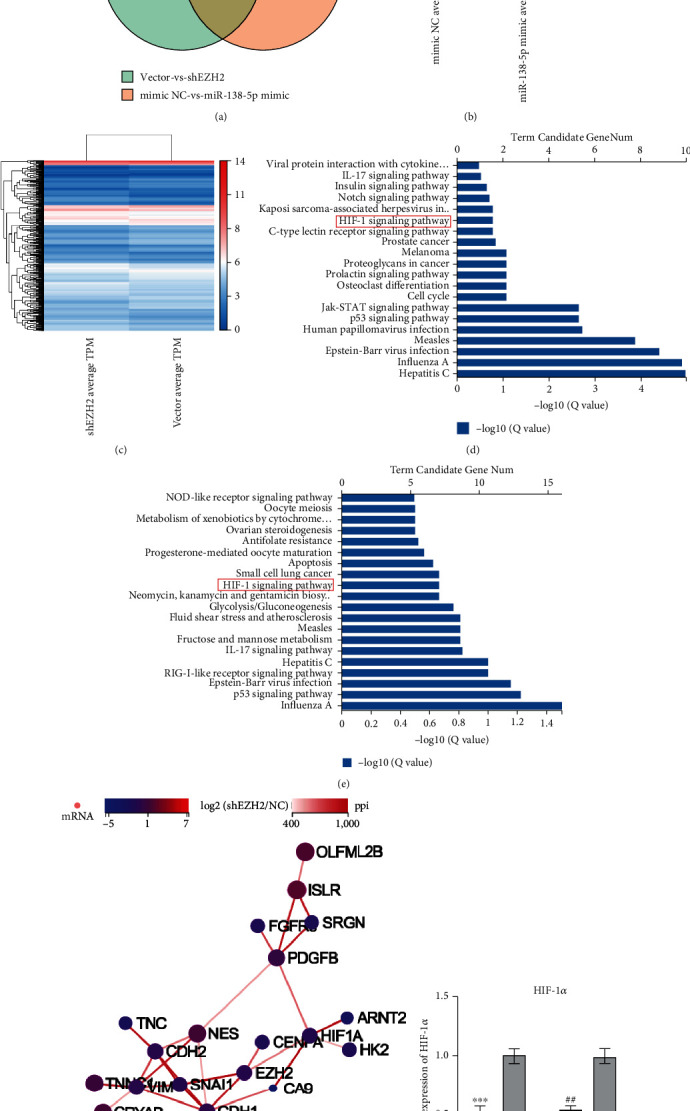
RNA-seq and verification. (a) Venn diagram showing the overlap of differentially expressed genes in two pair-wise comparisons (miR-138-5p mimic VS NC and HepG2-shEZH2 VS vector). (b–c) Heat maps of representative up and downregulated genes from HepG2 RNA-seq results. (d–e) The significant pathways enriched from the critical differential genes in KEGG analysis. (f) Network interaction diagram of representative and downregulated genes, EZH2, EMT, and HIF-1*α*-related genes from RNA-seq results. (g) In HepG2 and Hep3B cells, the expression detection of HIF-1*α* mRNA after transfection of miR-138-5p mimic by RT-qPCR.

HepG2 cells were treated with miR-138-5p mimic/NC or shEZH2/vector plasmid and then analyzed by RNA-Seq. The Venn diagram and heat map revealed 576 differential genes between HepG2-shEZH2/HepG2-vector groups (281 downregulated genes and 295 upregulated genes) and 108 differential genes between miR-138-5p mimic/NC groups (33 downregulated genes and 75 upregulated genes) (Figures 6(a)–6(c)). As determined by KEGG analysis, differential genes in both groups were enriched in the HIF-1 signaling pathway (Figures 6(d)–6(e)). Then, we plotted a network interaction diagram with EZH2 and HIF-1*α* as the main candidate genes, showing a potential regulatory relationship between EZH2 and HIF-1*α* (Figure 6(f)). We confirmed the expression of HIF-1*α* via RT-qPCR analyses. The HIF-1*α* expression in HepG2 and Hep3B cells was downregulated upon miR-138-5p mimic treatment (Figure 6(g)).

### 3.6. Dysregulation of EZH2/miR-138-5p Axis Affects EMT via HIF-1*α*

**Figure 7:**
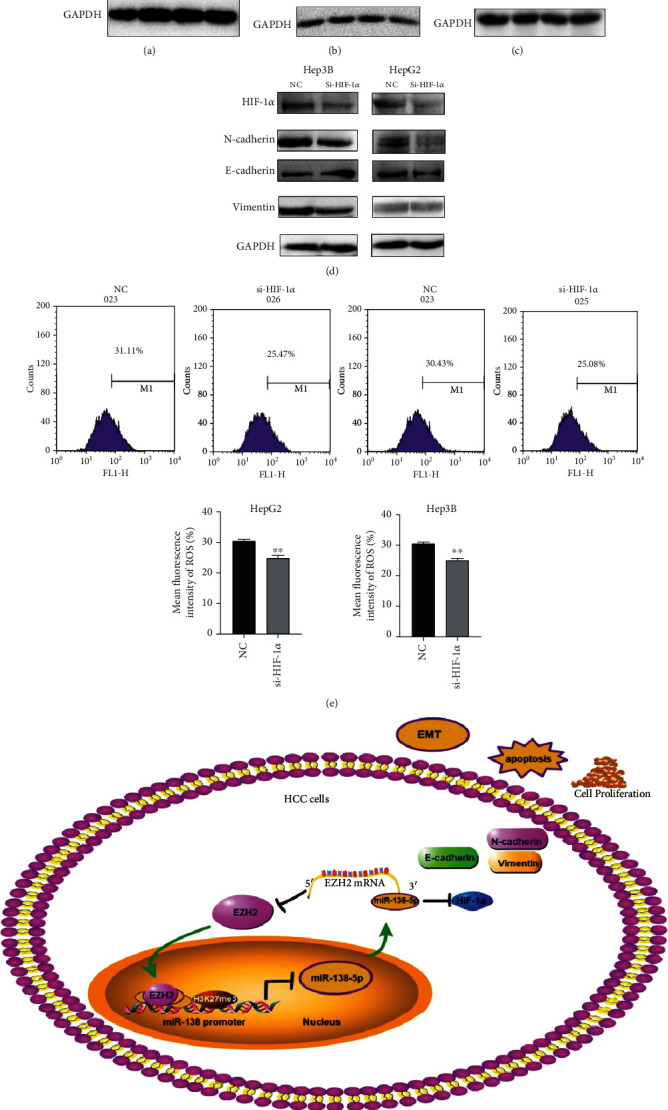
Dysregulation of EZH2/miR-138-5p axis affects EMT via HIF-1*α*. (a–c) In HepG2 and Hep3B cells, HIF-1*α* levels after transfection of miR-138-5p mimic were analyzed by Western Blot after radiation. (d) The effect of HIF-1*α* knockdown on the expression of EMT-related molecules by Western blotting (compared to NC group or vector group). (e) The level of ROS in HepG2 and Hep3B cells after transfection of siRNA of HIF-1*α*. (f) Proposed model for EZH2/miR-138 regulatory axis involved in EMT of HCC cells. ^∗^*p* < 0.05; ^∗∗^*p* < 0.01 compared to NC group.

Hypoxia is an environmental feature at the aggressive front of tumors, where EMT program takes place. Hypoxia-activated HIF-1*α* induces cancer EMT through multiple molecules and pathways, including epigenetic regulators and transcription factors/repressors [26]. To identify whether HIF-1 is involved in the regulation of EMT by this axis, we further analyzed the expression of HIF-1*α* in each group. Our results showed that protein level of HIF-1*α* was decreased after overexpressing miR-138-5p or inhibiting EZH2 after cells were exposed to 8 Gy radiation (Figures 7(a)–7(c)). The expression of HIF-1*α* was then reduced using siRNA in HepG2 and Hep3B cells. We found that the epithelial marker E-cadherin was upregulated, whereas the mesenchymal markers N-cadherin and Vimentin were downregulated (Figure 7(d)). Flow cytometry analysis also detected the change of intracellular ROS content after inhibiting HIF-1*α* expression. These data suggest that inhibition of HIF-1*α* expression can suppress the level of ROS (Figure 7(e)).

## 4. Discussion

This study investigated the role of the EZH2/miR-138-5p regulatory feedback loop in the post-radiation response of HCC. Multiple studies have shown a correlation between high EZH2 expression and poor prognosis in different cancers, including HCC [5, 27]. It has also been reported that EZH2 can protect glioma stem cells from radiation-induced cell death [28]. The high expression of EZH2 may be one of the mechanisms of bladder cancer cells in acquiring radiation resistance [29]. However, the role of EZH2 in the response of HCC cells to radiation is unclear. Here, we show that inhibition of EZH2 expression can restore the radiosensitivity of HCC cells. It is worth noting that several studies have reported upregulation of EZH2 expression in HCC, of which the underlying mechanism remains unknown. Also, since changes in miRNA expression appear to be a common feature of cancers, including HCC, inhibition of miRNAs targeting EZH2 may be associated with aberrant overexpression of EZH2. We here provided clear evidence that miR-138-5p negatively regulates EZH2 expression in HCC. Compared with tumor adjacent tissue, miR-138 was downregulated in HCC patient samples and negatively correlated with HCC survival. The negative correlation between miR-138 and EZH2 expression levels in HCC patients could be explained by the fact that EZH2 is a direct target of miR-138, as revealed by the luciferase assay. Similar to our findings, miRNA-associated EZH2 overexpression has also been reported in other tumors [30, 31].

Growing evidence suggests that EZH2 can also negatively regulate miRNAs through a feedback loop in tumors [32, 33]. In HCC, it has been reported that histone methylation can silence some tumor suppressor miRNAs and, in turn, lead to the upregulation of some oncogenes [34, 35]. Our data supported such a notion as miR-138 was shown to be epigenetically regulated by transcriptional suppression of its promoter region in HCC cells. Inhibition of EZH2 reduced H3K27me3 marker enrichment in the promoter region of miR-138-5p. These findings suggest a regulatory loop between the epigenetic silencing mechanism of miR-138-5p and EZH2 in HCC cells.

To verify the clinical relevance of EZH2 and the prognosis of HCC patients, we analyzed the TCGA database and found that patients with high EZH2 expression had significantly poorer survival. With increasing HCC grade, the expression of EZH2 was significantly increased. These data suggest that EZH2 upregulation is a predictor of poor prognosis. Therefore, anti-EZH2 therapy may serve as a promising strategy for the treatment of HCC. In view of the expression levels of EZH2 and miR-138-5p in liver cancer patients, we verified the changes in the radiosensitivity of HCC cells after changing the expression levels.

In addition, one of the most important findings of this paper is that overexpression of miR-138-5p in HCC cells can effectively enhance the radiosensitivity and reduce the ability of proliferation and apoptosis of HCC cells after radiation, and silencing of the EZH2 gene can achieve similar effects. These data suggest that low expression of miR-138-5p has an impact on HCC cell radiosensitivity by forming a negative feedback loop with EZH2, which is a direct and functionally relevant target of miR-138-5p. Similar to miR-138-5p overexpression, EZH2 inhibition by EZH2 shRNA induced apoptosis and cell cycle arrest at the G0/G1 stage.

This observation may suggest that a high level of EZH2 in HCC cells leads to an imbalance of cellular feedback loops leading to dysregulation of miR-138-5p, which affects cellular radiosensitivity. This feedback loop in the cell has been in a certain steady state, and its dynamic behavior may be influenced by external stimuli. After receiving an external stimulus, the feedback system can switch from one stable state to another in order to adapt to the external stimulus. Our results also suggest that overexpression of miR-138-5p or inhibition of EZH2 inhibits the migratory and invasive ability of HCC cells after radiation, at least in part through inhibition of the EMT pathway.

Our RNA-seq study found that the expression of HIF-1*α* was downregulated in miR-138-5p mimic group compared to the NC group, which is also downregulated in HepG2-shEZH2 in comparison to HepG2-vector. We also confirmed that HIF-1*α* was significantly downregulated in HepG2, in which miR-138-5p is overexpressed or EZH2 is silenced. Notably, in some cells, miR-138-5p can directly target HIF-1*α* and inhibit its expression [36, 37]. Studies have also reported that EZH2 can affect the expression of HIF-1*α* by downregulated miR-146a-5p [38]. These results suggest that dysregulation of EZH2/miR-138-5p axis could downregulate HIF-1*α* in direct or indirect pathway. Although the molecular mechanism mentioned above needs to be further elucidated, the above studies including our results further confirmed that HIF-1*α* is an important downstream target gene of EZH2/miR-138-5p axis. In a normal oxygen environment, HIF-1*α* is at a completely inactive level. Conversely, when cells are hypoxic, elevated HIF enters the nucleus and then upregulates many genes involved in cancer progression. High level of HIF-1*α* promotes cancer progression through multiple mechanisms, including angiogenesis, cell proliferation and survival, invasion and metastasis, and therapy resistance. It was also reported that HIF-1*α* could promote metastasis and EMT through increasing ROS expression level in cancer cells [39]. It has also been reported that, in HCC cells, hypoxia leads to increase on ROS and HIF-1*α* levels, which promotes the progression of EMT through the Hedgehog pathway [40]. We further confirmed the above studies, and we found that ROS level were reduced and EMT progression was inhibited after using siRNA to knockdown HIF-1*α*.

## 5. Conclusions

In conclusion, we determined EZH2 and miR-138-5p reciprocally regulate each other in HCC cells via a negative feedback loop, which contributes to the change of HIF-1*α* expression (Figure 7(f)). Our findings provide new insights into the mechanism of miR-138-5p/EZH2/HIF-1*α* pathway in the radiosensitivity of HCC. This study establishes EZH2/miR-138-5p axis as a potential predictor of poor prognosis and therapeutic target for HCC patients.

## Figures and Tables

**Table 1:** Sequences of the primers used for RT-qPCR and ChIP-qPCR.

Gene	Sequences (5′-3′)
miR-138-5p-F	TGACCGAGCTGGTGTTGTG
miR-138-5p-R	CAGAGCAGGGTCCGAGGTA
U6 snRNA-F	CGCTTCGGCAGCACATATAC
U6 snRNA-R	TTCACGAATTTGCGTGTCATC
EZH2-F	TGGTCTCCCCTACAGCAGAA
EZH2-R	TCATCTCCCATATAAGGAATGTTATG
GAPDH-F	GTGAAGGTCGGAGTCAACG
GAPDH-R	TGAGGTCAATGAAGGGGTC
HIF-1*α*-F	TAAGTTCTGAACGTCGAAAAGA
HIF-1*α*-R	CAGCATCCAGAAGTTTCCTC

ChIP-qPCR primer	
miR-138-5p-1-F	CCATTGTTTCTCTGCACCCC
miR-138-5p-1-R	GTGGCTCCTCCGGTTTTGAG
miR-138-5p-2-F	CACCTGGCTGGGAGTTCTT
miR-138-5p-2-R	CTATTGCAGTCCTGGTCCTC

**Table 2:** Cell survival curve fitting results.

Cell	Group	D_0_ (Gy)	n	Dq (Gy)	SF_2_	*R* ^2^
HepG2	miR-138-5p mimic	1.570	1.053	0.081	0.292	0.9998
	NC	2.259	1.001	0.002	0.403	0.9969
Hep3B	miR-138-5p mimic	1.416	1.233	0.297	0.291	0.9993
	NC	1.773	1.614	0.849	0.466	0.9985
HepG2	miR-138-5p mimic	1.591	2.957	1.725	0.626	0.9997
	NC	1.644	4.471	2.463	0.785	0.9991

## Data Availability

The data are presented within the paper. Additional raw data are available on request from the corresponding author.

## References

[1] Sung H., Ferlay J., Siegel R. L. (2021). Global cancer statistics 2020: GLOBOCAN estimates of incidence and mortality worldwide for 36 cancers in 185 countries. *CA: a Cancer Journal for Clinicians*.

[2] Kudo M., Trevisani F., Abou-Alfa G. K., Rimassa L. (2016). Hepatocellular carcinoma: therapeutic guidelines and medical treatment. *Liver Cancer*.

[3] Llovet J. M., Beaugrand M. (2003). Hepatocellular carcinoma: present status and future prospects. *Journal of Hepatology*.

[4] Gao S. B., Sun S. L., Zheng Q. L. (2015). Genetic alteration and misexpression of Polycomb group genes in hepatocellular carcinoma. *American Journal of Cancer Research*.

[5] Cai M. Y., Tong Z. T., Zheng F. (2011). EZH2 protein: a promising immunomarker for the detection of hepatocellular carcinomas in liver needle biopsies. *Gut*.

[6] Sudo T., Utsunomiya T., Mimori K. (2005). Clinicopathological significance of EZH2 mRNA expression in patients with hepatocellular carcinoma. *British Journal of Cancer*.

[7] Sasaki M., Ikeda H., Itatsu K. (2008). The overexpression of polycomb group proteins Bmi1 and EZH2 is associated with the progression and aggressive biological behavior of hepatocellular carcinoma. *Laboratory Investigation*.

[8] Cheng A. S., Lau S. S., Chen Y. (2011). EZH2-mediated concordant repression of Wnt antagonists promotes *β*-catenin-dependent hepatocarcinogenesis. *Cancer Research*.

[9] Huang B., Huang M., Li Q. (2018). MiR-137 suppresses migration and invasion by targeting EZH2-STAT3 signaling in human hepatocellular carcinoma. *Pathology, Research and Practice*.

[10] Shi Y., Yang X., Xue X. (2018). HANR promotes hepatocellular carcinoma progression via miR-214/EZH2/TGF-*β* axis. *Biochemical and Biophysical Research Communications*.

[11] Lee S. T., Li Z., Wu Z. (2011). Context-specific regulation of NF-*κ*B target gene expression by EZH2 in breast cancers. *Molecular Cell*.

[12] Xu K., Wu Z. J., Groner A. C. (2012). EZH2 oncogenic activity in castration-resistant prostate cancer cells is poly-comb-independent. *Science*.

[13] Li Z., Hou P., Fan D. (2017). The degradation of EZH2 mediated by lncRNA ANCR attenuated the invasion and metastasis of breast cancer. *Cell Death and Differentiation*.

[14] Cao Z., Wu W., Wei H., Zhang W., Huang Y., Dong Z. (2021). Downregulation of histone-lysine N-methyltransferase EZH2 inhibits cell viability and enhances chemosensitivity in lung cancer cells. *Oncology Letters*.

[15] Zeng T., Luo L., Huang Y., Ye X., Lin J. (2021). Upregulation of miR-138 increases sensitivity to cisplatin in hepatocellular carcinoma by regulating EZH2. *BioMed Research International*.

[16] Zhang D. Y., Sun Q. C., Zou X. J. (2020). Long noncoding RNA UPK1A-AS1 indicates poor prognosis of hepatocellular carcinoma and promotes cell proliferation through inter-action with EZH2. *Journal of Experimental & Clinical Cancer Research*.

[17] Harris A. L. (2002). Hypoxia--a key regulatory factor in tumour growth. *Nature Reviews. Cancer*.

[18] Rofstad E. K., Sundfør K., Lyng H., Tropé C. G. (2000). Hypoxia-induced treatment failure in advanced squamous cell carcinoma of the uterine cervix is primarily due to hypoxia-induced radiation resistance rather than hypoxia-induced metastasis. *British Journal of Cancer*.

[19] Chen J., Chen J., Huang J. (2019). HIF-2*α* upregulation mediated by hypoxia promotes NAFLD-HCC progression by activating lipid synthesis via the PI3K-AKT-mTOR pathway. *Aging (Albany NY)*.

[20] Moreno Roig E., Groot A. J., Yaromina A. (2019). HIF-1*α* and HIF-2*α* differently regulate the radiation sensitivity of NSCLC cells. *Cells*.

[21] Meijer T. W., Kaanders J. H., Span P. N., Bussink J. (2012). Targeting hypoxia, HIF-1, and tumor glucose metabolism to improve radiotherapy efficacy. *Clinical Cancer Research*.

[22] Yang W., Sun T., Cao J., Fan S. (2011). Hypoxia-inducible factor-1*α* downregulation by small interfering RNA inhibits proliferation, induces apoptosis, and enhances radiosensitivity in chemical hypoxic human hepatoma SMMC-7721 cells. *Cancer Biotherapy & Radiopharmaceuticals*.

[23] Chandrashekar D. S., Bashel B., Balasubramanya S. A. H. (2017). UALCAN: a portal for facilitating tumor subgroup gene expression and survival analyses. *Neoplasia*.

[24] Győrffy B. (2021). Survival analysis across the entire transcriptome identifies biomarkers with the highest prognostic power in breast cancer. *Computational and Structural Biotechnology Journal*.

[25] Lánczky A., Győrffy B. (2021). Web-based survival analysis tool tailored for medical research (KMplot): development and implementation. *Journal of Medical Internet Research*.

[26] Bao B., Azmi A. S., Ali S. (2012). The biological kinship of hy-poxia with CSC and EMT and their relationship with deregulated expression of miRNAs and tumor aggressiveness. *Biochimica et Biophysica Acta*.

[27] Eich M. L., Athar M., Ferguson J. E., Varambally S. (2020). EZH2-targeted therapies in cancer: hype or a reality. *Cancer Research*.

[28] Kim S. H., Joshi K., Ezhilarasan R. (2015). EZH2 protects glioma stem cells from radiation-induced cell death in a MELK/FOXM1-dependent manner. *Stem Cell Reports*.

[29] Zhang X., Ma X., Wang Q., Kong Z. (2022). EZH2 targeting to improve the sensitivity of acquired radio-resistance bladder cancer cells. *Translational Oncology*.

[30] Li Y., Zhou H. C., Zhang Y., Huang H. (2020). MicroRNA-625-3p inhibits gastric cancer metastasis through modulating EZH2. *European Review for Medical and Pharmacological Sciences*.

[31] Sun X. B., Chen Y. W., Yao Q. S. (2021). Mi-croRNA-144 suppresses prostate cancer growth and metastasis by targeting EZH2. *Technology in Cancer Research & Treatment*.

[32] Wang L., Zhang X., Jia L. T. (2014). C-Myc-mediated epigenetic silencing of MicroRNA-101 contributes to dysregulation of multiple pathways in hepatocellular carcinoma. *Hepatology*.

[33] Zhuang C., Wang P., Huang D. (2016). A double-negative feedback loop between EZH2 and miR-26a regulates tumor cell growth in hepatocellular carcinoma. *International Journal of Oncology*.

[34] Hou J., Lin L., Zhou W. (2011). Identification of miRNomes in human liver and hepatocellular carcinoma reveals miR-199a/b-3p as therapeutic target for hepatocellular carcinoma. *Cancer Cell*.

[35] Zhang X., Zhang X., Wang T. (2018). MicroRNA-26a is a key regulon that inhibits progression and metastasis of c-Myc/EZH2 double high advanced hepatocellular carcinoma. *Cancer Letters*.

[36] Gai Y. S., Ren Y. H., Gao Y., Liu H. N. (2020). Astaxanthin protecting myocardial cells from hypoxia/reoxygenation injury by regulating miR-138/HIF-1*α* axis. *European Review for Medical and Pharmacological Sciences*.

[37] Mei H., Xian H., Ke J. (2021). LncRNA-MCM3AP-AS1 promotes the progression of infantile hemangiomas by increasing miR-138-5p/HIF-1*α* axis-regulated glycolysis. *Frontiers in Molecular Biosciences*.

[38] Ni S., Yang B., Xia L., Zhang H. (2021). EZH2 mediates miR-146a-5p/HIF-1*α* to alleviate inflammation and glycolysis after acute spinal cord injury. *Mediators of Inflammation*.

[39] Xu W. N., Yang R. Z., Zheng H. L., Jiang L. S., Jiang S. D. (2020). NDUFA4L2 regulated by HIF-1*α* promotes metastasis and epithelial-mesenchymal transition of osteosarcoma cells through inhibiting ROS production. *Frontiers in Cell and Development Biology*.

[40] Liu Z., Tu K., Wang Y. (2017). Hypoxia accelerates aggressiveness of hepatocellular carcinoma cells involving oxidative stress, epithelial-mesenchymal transition and non-canonical hedgehog signaling. *Cellular Physiology and Biochemistry*.

